# C-851-02. Assessing the Impact of Frailty on Surgical Decision-Making in Older Adults with Burn Injury

**DOI:** 10.1093/jbcr/irag033.054

**Published:** 2026-04-06

**Authors:** Nina Putnam, Shawn Tejiram, Kathleen S Romanowski, Lauren B Nosanov, David M Hill, Colette Galet, Dhaval Bhavsar, Alisa Savetamal, Alexandra M Lacey, Mark J Johnston, Lucy Wibbenmeyer, Sara Higginson, Nicole M Kopari, Deepak K Ozhathil

**Affiliations:** University of California Davis Health, Sacramento, CA; MedStar Washington Hospital Center, Washington, DC; University of California Davis Burn Center & Shriners Hospital for Children, Sacramento, CA; Emory University School of Medicine, Atlanta, Georgia; Firefighters Regional One Burn Center, Memphis, TN; University of Iowa, Iowa City, IA; The University of Kansas Health System, Kansas City, KS; Bridgeport Hospital/Yale New Haven Health, Bridgeport, CT; Regions Hospital, Saint Paul, MN; Regions Hospital, Saint Paul, MN; University of Iowa Health Care, Iowa City, IA; Shriners Children's - Ohio, Dayton, OH; Leon S. Peters Burn Center, Fresno, California; Akron Children's Hospital, Akron, OH

## Abstract

**Introduction:**

Older adults make up a growing proportion of burn patients and present unique challenges requiring tailored approaches. Frailty, defined as decreased physiologic reserve and vulnerability to stressors, has emerged as an important predictor of outcomes in this population. In burn care, frailty may influence decisions about candidacy, timing, and extent of surgery, but its role remains unclear. We aimed to examine the association between frailty and operative decision-making in older adult burn patients.

**Methods:**

Following IRB approval, we conducted a retrospective multicenter cohort study of patients ≥60 years admitted to 12 burn centers (1/2017–12/2019). Demographics, injury characteristics, and operative variables were collected. Frailty was measured with the Canadian Study of Health and Aging Clinical Frailty Scale and categorized as fit (< 4), prefrail (=4), or frail (>4). Univariate and multivariate analyses were performed to assess associations between frailty and operative decision-making. *p*<.05 was significant.

**Results:**

Of 1478 patients with clinical frailty scores collected, 767 underwent surgery. Surgical patients were more often fit (371, 59.3%) than prefrail (190, 54.3%) or frail (206, 41%) (*p*<.0001). Among males, surgery was more common in the fit (274, 73.9%) and prefrail (138, 72.6%) groups than the frail (122, 59.2%). Frail patients were older (70 [64–79]) compared with prefrail (70 [64–76]) and fit (66 [62–74]) (*p*<.001). Injury type differed, with most patients sustaining flame or flash burns (*p*=.007), but TBSA and inhalation injury did not vary by frailty. Frail patients had more operations (1 [1–3]) than prefrail (1 [1–2]) and fit (1 [1–2]) (*p*=.005). Time from injury to surgery was longest in prefrail patients, followed by frail, and shortest in fit (5 [3–10.3] vs. 4 [2–9] vs. 4 [2–7], *p*=.004). Prefrail patients were most likely to undergo complete excision and grafting in one operation (50.5% vs. 49.6% vs. 39.8%, *p*=.04). Frail patients had greater allograft use (32%) compared with prefrail (30.5%) and fit (21.3%) (*p*=.007). Dermal substitute use and graft failure did not differ by frailty category. On multivariate analysis, allograft use was independently associated with TBSA (OR 1.03 [1.02–1.05]) and frailty (fit vs. frail OR 0.51 [0.34–0.76]). Treatment in a single operation was associated with TBSA (OR 0.95 [0.94–0.97]) and frailty (fit vs. frail OR 1.67 [1.16–2.38]; prefrail vs. frail OR 1.66 [1.10–2.52]).

**Conclusions:**

Frailty influences operative care in older burn patients. Fit patients undergo surgery sooner and in fewer stages, while frail patients require more procedures and more often receive allograft.

**Applicability of Research to Practice:**

Frailty assessment at admission provides prognostic and operative planning value and should be integrated into burn care to support surgical decision-making and patient-centered care.

**Funding for the Study:**

N/A.

